# The associations between psychosocial aspects and TMD-pain related aspects in children and adolescents

**DOI:** 10.1186/s10194-016-0622-0

**Published:** 2016-04-05

**Authors:** Amal Al-Khotani, Aron Naimi-Akbar, Mattias Gjelset, Emad Albadawi, Lanre Bello, Britt Hedenberg-Magnusson, Nikolaos Christidis

**Affiliations:** Orofacial Pain and Jaw Function, Department of Dental Medicine, Karolinska Institutet, SE-141 04 Huddinge, Sweden; Scandinavian Center for Orofacial Neurosciences (SCON), Huddinge, Sweden; Cariology, Department of Dental Medicine, Karolinska Institutet, SE-141 04 Huddinge, Sweden; Dental Speciality Center, Ministry of Health, Jeddah, Saudi Arabia; Pediatric Dentistry and Orthodontics Department, College of Dentistry, King Saud University, Riyadh, Saudi Arabia; Department of Clinical Oral Physiology at the Eastman Institute, Stockholm Public Dental Health (Folktandvården SLL AB), SE-113 24 Stockholm, Sweden

**Keywords:** Temporomandibular disorders, Children, Adolescents, Psychosocial, Pain

## Abstract

**Background:**

Temporomandibular disorders (TMD) in children and adolescents is prevalent with pain as a common component, and has a comorbidity with psychosocial problems such as stress, depression, anxiety as well as somatic complaints. Therefore, the aim of the study was to investigate if psychosocial problems in children and adolescents are associated with TMD with pain (TMD-pain) and TMD without pain (TMD-painfree) when compared to children and adolescents without TMD.

**Methods:**

This cross-sectional study consisted of 456 randomly selected children and adolescents, enrolled from 10 boy’s- and 10 girl’s- schools in Jeddah, between 10 and 18 years of age. On the examination day, prior to the clinical examination according to Research Diagnostic Criteria for TMD Axis I and II, the participants first answered two validated questions about TMD pain, and after that the Arabic version of the Youth Self Report scale. According to their clinical examination and diagnosis the participants were divided into three groups; non-TMD group, TMD-pain group, and TMD-painfree group.

**Results:**

The TMD-pain group presents a higher frequency of the internalizing problems anxiety, depression and somatic complaints than non-TMD group (*p* < 0.05). Regarding externalizing problems the only significant association found was for aggressive behavior in the TMD-pain group (*p* < 0.05). The TMD-pain group also shows a higher frequency of social problems than the non-TMD group. However, no such difference was found when compared to the TMD-painfree group. There was also a significant association with a higher frequency of thought problems in the TMD-pain group (*p* < 0.05). The children’s and adolescents’ physical activities were within border line clinical range for all three groups, whereas the social competence was within the normal range. There were no significant associations between any of the groups in this respect.

**Conclusions:**

TMD-pain in children and adolescents does not seem to affect the social activities. However, TMD-pain seem to have a strong association to emotional, behavior and somatic functioning, with higher frequencies of anxiety, depression, somatic problems, aggressive behavior and thought problems, than children and adolescents without TMD-pain. With respect to the biopsychosocial model the present study indicates that there are significant associations to psychosocial, somatic and behavioral comorbidities and TMD-pain in children and adolescents in the Middle East region.

## Background

Temporomandibular disorders (TMD) is multifactorial broad-band term that embraces chronic pain conditions and dysfunction (both painful and painfree dysfunctions) in the orofacial region affecting the masticatory muscles, the temporomandibular joints (TMJ) and their associated structures. TMD is often associated with restricted mouth opening capacity, pain upon chewing, muscle soreness and headache. Although TMD is not life threatening, it affects quality of life considerably [1].

Recent studies indicate that the prevalence of diagnosed TMD in children and adolescents is increasing, and reaching as many as 27 % of the children and adolescents in the general population (personal communication, 2016) and approximately 30 % in a clinical setting [2, 3]. In older studies from 15 years ago the prevalence was approximately 7–14 % [4, 5]. More than 80 % of the children and adolescents diagnosed with TMD were complaining of orofacial pain (personal communication, 2016).

Further, it is well known that psychosocial problems in children and adolescents are more frequent than in the past, and also that these problems have a negative effect on children’s wellbeing [6]. Further, according to the biopsychosocial theory, somatic pain is directly associated with psychological, biological as well as social perspectives. This association often continues to adulthood, consequently with a risk of extension of both somatic pain and psychosocial burdens [7, 8]. As for more general pain conditions, the biopsychosocial theory can also be applied for TMD. Several studies have shown that patients suffering from TMD also reported different psychosocial problems [9–11], somatic complaints [12], and functional impairments [13] at various intensities. Other studies have shown strong associations between TMD and emotional stress, depression, anxiety and somatic complaints [2, 14, 15]. Similarly, it has been shown that children and adolescents suffering from pain often are diagnosed with psychological conditions, including depressive disorders, and a long-term diminished quality of life [16].

Two earlier studies have investigated the relationship between TMD and behavior as well as psychosocial functioning in children and adolescents [17, 18], using the Youth Self Report scale (YSR; ASEBA School-Age Forms & Profiles) [19], or part of it. The YSR is a reliable and validated scale that evaluates competencies, psychosocial, and somatic problems in many dimensional relationships in the younger ages [20]. According to Achenbach’s guidelines, YSR can be applied in children and adolescents with a mental age of 10 but not exceeding the age of 18 [19]. One of the studies presented the psychosocial functioning among patients with TMD reporting pain [17, 18], whereas in the other study, participants who reported pain (from a questionnaire) had the chance to be examined for TMD [17, 18]. None of the studies found any association between the psychosocial functioning in children and adolescents with TMD with pain and children and adolescents without TMD as well as between children and adolescents with TMD without pain and children and adolescents without TMD.

Taken together, there is a high prevalence of TMD in children and adolescents with pain as a common component with a comorbidity with psychosocial problems such as stress, depression, anxiety as well as somatic complaints. With this in mind, the hypothesis of the present study was that psychosocial problems in children and adolescents are associated with a diagnosis of TMD with pain (TMD-pain) than a diagnosis of TMD without pain (TMD-painfree). Therefore, the aim of the study was to investigate if psychosocial problems in children and adolescents are associated with TMD-pain and TMD-painfree when compared to a children and adolescents without TMD.

## Methods

The present study was a cross sectional study, carried out on children and adolescents among the general population from a major city in Saudi Arabia (Jeddah). It was approved by the local ethical committee at the Department of Medical Study and Research, Ministry of Health, Jeddah, Saudi Arabia. Prior to inclusion all participants received both written and verbal information, and gave their verbal and written consent. The study followed the guidelines of the Declaration of Helsinki.

### Participants

The education in Saudi Arabia is based on single-sex schools. Hence, in order to obtain a representative sample of the entire Jeddah city, the selection was based on a predefined set of schools, as clustered by the ministry of education. Therefore, the city of Jeddah was divided into five regions (North, South, East, West, and Central). From each region two schools with boys and two schools with girls were randomly selected. The randomization from each region was performed by a researcher (NC), who did not participate in data collection, with an internet-based application (www.randomization.com). Further, from each school one class, with an average of 30 pupils, was also randomly selected using simple sampling method; hence, the school classes’ titles are drawn from a bucket by dental assistant not participating in the data collection.

**Fig. 1:**
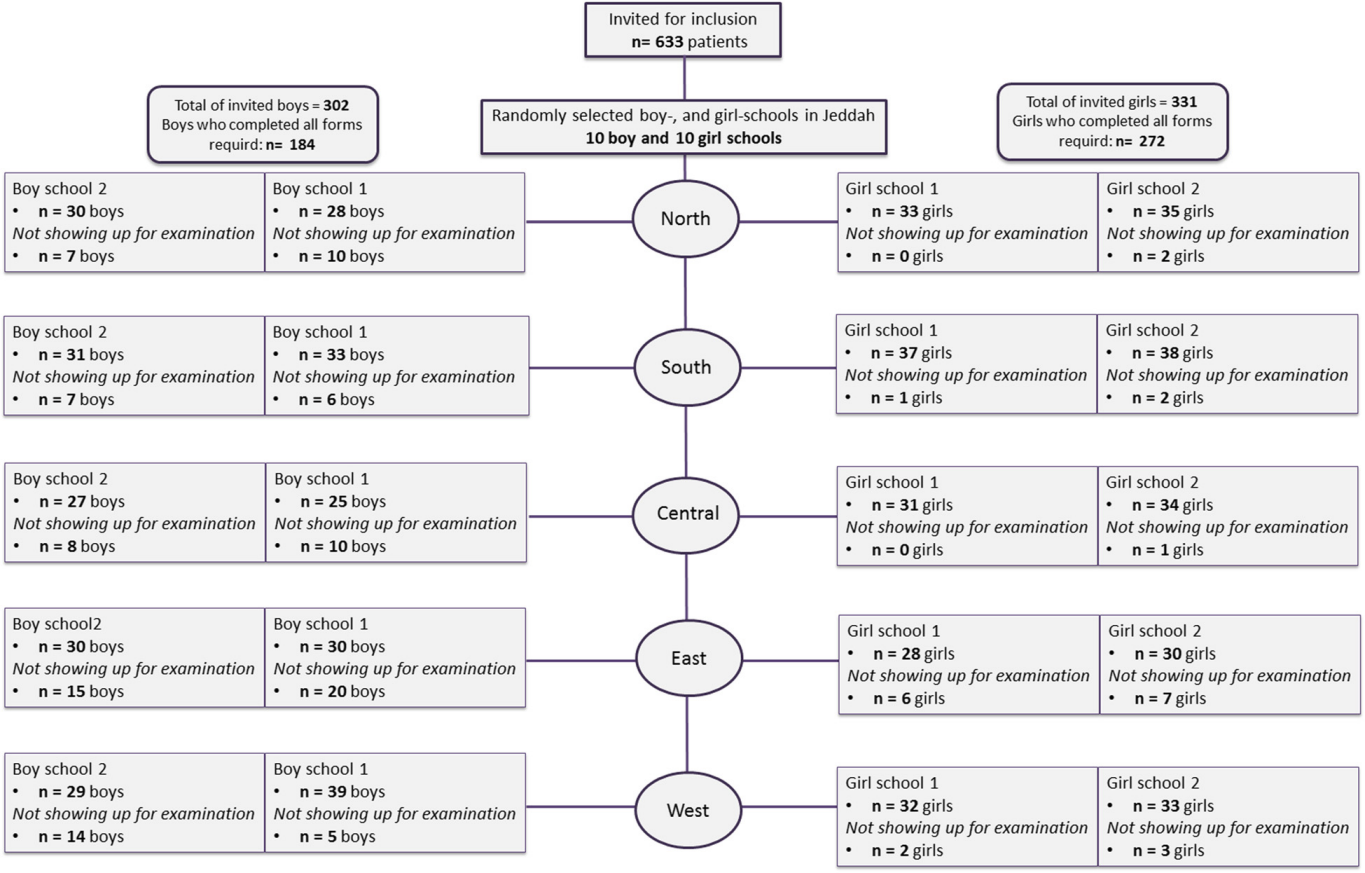
Flowchart of the participating children and adolescents. Flowchart of the 456 participating children and adolescents from the general population of the city of Jeddah, Saudi Arabia

In order to achieve generalizable results, all possible participants were invited, thus the present study did not have any exclusion criteria. Out of the 633 children and adolescents who were invited to participate, 509 voluntarily agreed to participate, and 456 completed all questionnaires and participated in the clinical examination, aged between 10 and 18 years, as shown in Fig. 1.

### Study protocol

This study presents the Axis II of the Research Diagnostic Criteria for TMD (RDC/TMD). Axis I of the RDC/TMD is presented in another study (not yet published), thus, the phrase “personal communication, 2016” will be used in this study in order to refer to the previous findings from that study. According to the outcomes of the Axis I in the RDC/TMD examination (personal communication, 2016) the children and adolescents were divided into three groups; a) non-TMD which includes children and adolescents without any TMD diagnosis; b) TMD-pain which includes children and adolescents having a TMD diagnosis with pain; and c) TMD-painfree which includes children and adolescents having a TMD diagnosis without pain”.

Due to cultural considerations, there was one protocol for boys and one for girls. All girls were examined in the school nurse’s room using a mobile dental chair. However, all boys were invited to be examined at a dental clinic of the primary health care center for each region. In order to minimize the impact of the surroundings during the examination equal equipment were used at both examination facilities.

#### Protocol for girls

One day before the clinical examination, proper information about the purpose of the study and a brief explanation of the questionnaires was presented to all girls and their parents, and the RDC/TMD history questionnaire was distributed in sealed envelopes. From the RDC/TMD questionnaire, demographic data (including ethnic and socioeconomic background information), medical history, presence of oral parafunctions, headache, previous trauma to the face, and use of oral appliances was retrieved. In addition, the scores for the Graded Chronic Pain Scale (GCPS) included in the questionnaire were also retrieved.

On the day of examination, the girls were asked to fill in the official Arabic version of the Youth Self Report (YSR), licensed from ASEBA/Research Center for Children, Youth & Families, university of Vermont, Burlington, USA. After completing this questionnaire, each participant was asked two validated questions about the presence of orofacial pain (TMD-pain) [21, 22]; 1) *“Do you have pain in the temple, face, temporomandibular joint, or jaws once a week or more?”* 2) *“Do you have pain when you open your mouth wide or chew once a week or more?”*. Finally, the clinical examination of the temporomandibular region according to the RDC/TMD Axis I protocol was performed by one examiner (A A-K), trained in this procedure by an orofacial pain specialist (Malin Ernberg; calibrated to a gold-standard examiner (Thomas List)).

#### Protocol for boys

One appointment was offered to each presumable participant and proper information about the purpose of the study and a brief explanation of the questionnaires was presented to all boys and their parents. As for the girls the RDC/TMD questionnaire including the GCPS, the YSR questionnaire and the demographic data (including ethnic and socioeconomic background information), medical history, presence of oral parafunctions, headache, previous trauma to the face, and use of oral appliances was retrieved before the clinical examination. The boys were accompanied by a parent/guardian to the clinic but the parents were asked to wait outside the clinical room. However, if the parent insisted to attend together with their child, they were asked to remain passive during the entire session.

### Emotional/behavior and somatic functioning

The RDC/TMD is a widely used dual diagnostic tool (Axis I and II), also reliable to be used in children and adolescents [21]. Axis I is used in order to diagnose TMD and Axis II in order to identify psychosocial and somatic symptoms using the Symptom Checklist-90-Revised (SCL-90-R). However, the SCL-90-R is not validated for children and adolescents younger than 13 years of age [23], and was therefore replaced with YSR, which is a scale that shares the same purpose but for children and adolescents in all school ages [20].

The emotional and behavior functioning were assessed using the YSR [20]. The YSR comprises of two main domains: 1) Problem Checklist, and 2) Social competence. The Problem Checklist contains 112 problem statements. As in a previous study regarding TMD pain in adults in Saudi Arabia, 3 statements about sexual problems were removed in the current study, due to cultural considerations [24]. The statements of the Problem Checklist explicate the major clusters: anxiety, depression, somatic complain, aggressive disorders, as well as social and attention problems. The major clusters are grouped into 3 subscales; a) broad-band internalizing and externalizing, b) eight narrow-band syndromes, c) DSM-oriented scales [20]. The eight narrow band syndromes are; 1) Anxious/Depressed, 2) Withdrawn/Depressed, 3) Somatic Complaints, 4) Social problems, 5) Thought problems, 6) Attention problems, 7) Rule-breaking behavior, and 8) Aggressive behavior. The DSM-oriented scales include; I) Affective problems, II) Anxiety problems, III) Somatic problems, IV) Attention Deficit/Hyperactivity problems, V) Oppositional Defiant problems, and VI) Conduct problems. Each statement is rated as 0 (not true), 1 (somewhat or sometimes true), or 2 (very true or often true) The YSR is reliable and valid [20]. Both for the data entry (scores) and for proper data grouping into the subscales, a licensed software scoring program (ASEBA™ version 9.1) was used. As a result, percentiles and T-scores are presented for all subscales and syndromes. The normal T-score range for all syndromes is 50–64, the border line clinical range is 65–69, while the clinical range is 70–100.

### Physical activities and social competence

The second domain of YSR, i.e., the Social competence, comprises of seven statements which cover three areas; social relations, physical activities, and the mean of self-reported academic performance [20]. Also for this domain the licensed software scoring program (ASEBA™ version 9.1) was used. As a result, percentiles and T-scores are presented for all activities and social competences. The normal T-score range is 36–65, the border line clinical range is 32–35, while the clinical range is 20–31.

The Graded Chronic Pain Scale (GCPS) [25] is included in the RDC/TMD Axis II [26]. The GCPS severity scale is divided into two parts. The first part is used to assess characteristic pain intensity and the second part limitations in physical functioning due to pain, including disability days. The disability days comprises of four grades (0–3) where 0 = 0–6 disability days, 1 = 7–14 disability days, 2 = 15–30 disability days, and 3 = 31+ disability days. However, when assessing physical functioning disability points (DP 0–6) is combined with pain intensity (0–100) as follows: Grade 0 = no TMD-pain in the previous 6 months; Grade I = low disability (<3 DP) and low intensity pain (<50); Grade II = low disability (<3 DP) and high intensity pain (>50); Grade III = high disability, moderately limiting (3–4 DP regardless of pain intensity); Grade IV = high disability, severely limiting (5–6 DP regardless of pain intensity).

### Statistics

Depending on the diagnoses that are presented in a previous study (personal communication, 2016), 456 participants were divided into three groups; non-TMD, TMD-pain and TMD-painfree. According to the power calculation 450 children and adolescents were necessary to detect true odds ratios for disease of 0.538 up to 1.860 with a power of 90 % and a significance level of 0.05, but 633 were invited due to the risk of children and adolescents not showing up for examination.

The descriptive statistics are presented as mean (SD) and median (IQR) depending on distribution of data, and also frequencies (%). To analyze differences in T-scores between the three groups the median score was modeled using quantile regression. In the model not adjusted for potential confounding factors TMD groups was included as dichotomous dummy variables with non-TMD as the reference group. The multivariate model adjusting for potential confounding factors also included sex (male/female), age (10–13 years/14–18 years), as well as Saudi Arabian nationality (yes/no) as dichotomous variables. Family income was modeled as a dichotomous dummy variables and it included 3 categories (below average/average/above average) which was based on the average income in Saudi Arabia for the year 2013 (15 000 SR/month) (www.cdsi.gov.sa). Subgroup analyzes stratified on sex and age groups separately were also performed with the stratification variable excluded from the model. *P*-values were based on 100 bootstrap samples. All analyzes were performed in STATA 12 SE. *P*-values lower than 0.05 and confidence intervals not including 0 were considered statistically significant.

## Results

### Study population

The demographic characteristics for all children and adolescents are presented in Table 1. They were categorized into one of three groups; a) the non-TMD group: all children and adolescents with no definite diagnosis, b) the TMD-painfree group: children and adolescents diagnosed with either osteoarthrosis and/or disc displacement with or without reduction, and c) TMD-pain group: children and adolescents diagnosed with either myofascial pain with or without limited mouth opening and/or arthralgia and/or osteoarthritis. Of note, none of the children was diagnosed with disc displacement with reduction (personal communication, 2016). There were no differences among the groups in regards to demographic characteristics nor when medical and oral health were taken into consideration (personal communication, 2016). There were further no differences when taking the ethnic origin into consideration, i.e., between the Saudi and the Non-Saudi participants, neither in the background data nor in any of the outcomes.

**Table 1:** Demographic data from 456 randomly selected children and adolescents in the general population of the city of Jeddah, Saudi Arabia

	Non-TMD n (%)	TMD-painfree n (%)	TMD-pain n (%)
Individuals	332 (72.8 %)	26 (5.7 %)	98 (21.5 %)
Age			
Mean (SD)	13.9 (2.3)	14.6 (2.3)	14.2 (2.4)
Min-max	10–18	12–18	11–18
10–13 years	177 (53.3 %)	10 (38.5 %)	48 (49 %)
14–18 years	155 (46.7 %)	16 (61.5)	50 (51 %)
Sex			
Boys	138 (41.6 %)	11 (42.3 %)	35 (35.7 %)
Girls	194 (58.4 %)	15 (57.7 %)	63 (64.3 %)
Ethnic origin			
Saudi Arabia	217 (65.4 %)	13 (50 %)	61 (62.2 %)
Non-Saudi^a^	115 (34.6 %)	13 (50 %)	37 (37.8 %)
Parental income			
Below average	172 (53.4 %)	11 (45.8 %)	50 (51.6 %)
Average	109 (33.9 %)	11 (45.8 %)	29 (30 %)
Above average	41 (12.7 %)	2 (8.3 %)	18 (18.6 %)

^a^Middle East, Gulf Area and Africa

### Emotional/behavior and somatic functioning

#### Broad band internalizing and externalizing scale and narrow-band syndrome scale

The mean and median range of T-scores were within normal range in all three groups (50–64) for all syndromes of the narrow-band syndrome scale. In the unadjusted analysis the TMD-pain group presents significant associations with an increase in T-scores of internalizing problems, i.e., Anxious/Depressed, Withdrawn/Depressed and Somatic Complaints, when compared to the non-TMD group (*p* < 0.05). In the adjusted analysis, however, the TMD-pain group presents a significant association with the internalizing problems Withdrawn/Depressed as well as with Somatic Complaints (*p* < 0.05), as shown in Table 2.

**Table 2:** Associations between TMD and internalizing problems, externalizing problems, social, thought and attention problems. Regression coefficients are presented with 95 % confidence intervals retrieved from quantile regression analysis

	Syndromes	Non-TMD	TMD-painfree	TMD-pain
Internalizing problems	Anxious/Depressed			
	Unadjusted Coeff.	ref	−2	4^a^
	95 % CI		(−8.2)–4.2	1.2–6.6
	Adjusted Coeff.	ref	−4	2
	95 % CI		(−8.2)–0.2	(−1)–5
	Withdrawn/Depressed			
	Unadjusted Coeff.	ref	−1	3^a^
	95 % CI		(−4.5)–2.5	1.1–5
	Adjusted Coeff.	ref	−1	3^a^
	95 % CI		(−4.4)–2.4	0.7–5.2
	Somatic Complaints			
	Unadjusted Coeff.	ref	−1	5^a^
	95 % CI		(−4.1)–2.1	0.9–9.1
	Adjusted Coeff.	ref	−1	5^a^
	95 % CI		(−4.4)–2.4	1.7–8.3
	Social Problems			
	Unadjusted Coeff.	ref	−3	4^a^
	95 % CI		(−6.4)–0.4	(−1.9)–6.1
	Adjusted Coeff.	ref	−3	3^a^
	95 % CI		(−6.6)–0.6	0.7–5.3
	Thought Problems			
	Unadjusted Coeff.	ref	1	3^a^
	95 % CI		(−1.7)–3.7	1.3–4.7
	Adjusted Coeff.	ref	0	1
	95 % CI		(−2.2)–2.2	(−0.7)–2.7
	Attention Problem			
	Unadjusted Coeff.	ref	−1	2
	95 % CI		(−4.2)–2.2	(−0.8)–4.8
	Adjusted Coeff.	ref	−0.5	1.5
	95 % CI		(−3.7)–2.7	(−0.6)–3.6
Externalizing problems	Rule-Breaking Behavior			
	Unadjusted Coeff.	ref	−1	0
	95 % CI		(−2.8)–0.8	(−1.5)–1.5
	Adjusted Coeff.	ref	−1	0
	95 % CI		(−2.8)–0.8	(−1.3)–1.3
	Aggressive Behavior			
	Unadjusted Coeff.	ref	1	3^a^
	95 % CI		(−2.6)–4.6	0.2–5.8
	Adjusted Coeff.	ref	0	2
	95 % CI		(−2.9)–2.9	(−0.2)–4.2

^a^ = significant difference between TMD-pain group and TMD-painfree (*p* < 0.05)

Both unadjusted analysis and adjusted for age, sex, ethnic origin and parental income are presented

In the subgroup analyzes stratified on age there was a stronger association with increase in T-score for internalizing problems Anxious/Depressed and TMD-pain in the younger age group (10–13 years) (Coefficient = 5; 95 % CI: 0.9–9.1) compared to the older age group (14–18 years) (coefficient = 1; 95 % CI: − 3.1–5.1). Both retrieved from adjusted analyzes (*p* < 0.05).

Regarding externalizing problems the only significant association found was for Aggressive Behavior in the TMD-pain group according to the unadjusted analysis (*p* < 0.05), Table 2.

Further, the TMD-pain group showed a significant increase in Social problems than the non-TMD group both in the unadjusted and adjusted analysis (*p* < 0.05), shown in Table 2. When the internalized syndromes of the narrow-band syndrome scales were analyzed s stratified on sex, there were stronger associations with increased T-scores and TMD-pain among boys than girls, Anxious/Depressed (Coefficient = 5; 95 % CI: 0.4–9.6), Somatic Complaints (Coefficient = 10; 95 % CI: 4.9–15.1) (*p* < 0.001), and Attention Problem (Coefficient = 4; 95 % CI: 1.3–6.7) (*p* < 0.05) (presented coefficients are for boys). Further, there was more increased T-score associated with TMD-pain in the narrow-band syndrome Anxious/Depressed in the younger age group (10–13) compared to the older (10–13) (Coefficient =5; 95 % CI: 1.0–9.1) (*p* < 0.05). Finally, there was also a significant association of Thought problems in the TMD-pain group with the unadjusted analysis (*p* < 0.05). This association was not found in the adjusted analysis, also Table 2.

#### DSM-oriented scales

Table 3 shows that children and adolescents in the TMD-pain group reported presence of affective, anxiety, and somatic problems in the DSM-oriented scales significantly compared to non-TMD in both the unadjusted and adjusted analyses (*p* < 0.05). This significance was not found when compared to the TMD-painfree group. When the DSM-oriented scales were analyzed separately for age groups and sex there was a significantly increased risk to develop Affective Problems (Coefficient = 5; 95 % CI: 1.0–9.0), and Anxiety Problems (Coefficient = 3; 95 % CI: 0.7–5.3) (*p* < 0.05) in the younger age group (10–13) compared to the older age group (14–18) with TMD-pain. Further, a significant increase in Attention Deficit/Hyperactivity Problems T-scores (Coefficient =4; 95 % CI: 1.7–6.3) and Oppositional Defiant Problems (Coefficient =2; 95 % CI: 0.3–3.7) associated with TMD-pain in boys than girls. Further, there is also an increase Oppositional Defiant Problems (Coefficient =2; 95 % CI: 0.2–3.8) (*p* < 0.05) associated with TMD-pain in older age group (14–18) than younger age group (10–13).

**Table 3:** Associations between TMD and DSM-Oriented scale: Regression coefficients are presented with 95 % confidence intervals retrieved from quantile regression analysis

	Non-TMD	TMD-painfree	TMD-pain
Affective problems			
Unadjusted Coeff.	ref	3	4^a^
95 % CI		(−0.1)–6.1	2.2–5.8
Adjusted Coeff.	ref	1	3^a^
95 % CI		(−2.8)–4.8	0.5–5.5
Anxiety problems			
Unadjusted Coeff.	ref	0	3^a^
95 % CI		(−2.7)–2.7	0.3–5.7
Adjusted Coeff.	ref	−1	4^a^
95 % CI		(−3.9)–1.9	1.3–6.7
Somatic problems			
Unadjusted Coeff.	ref	−1	5^a^
95 % CI		(−2.6)–0.6	3.2–6.8
Adjusted Coeff.	ref	−1	3.5^a^
95 % CI		(−2.8)–0.8	1.3–5.7
Attention deficit/hyperactivity problems			
Unadjusted Coeff.	ref	0	1
95 % CI		(−1.5)–1.5	(−0.7)–2.7
Adjusted Coeff.	ref	0	1
95 % CI		(−1.5)–1.5	(−0.4)–2.4
Oppositional defiant problems			
Unadjusted Coeff.	ref	0	0
95 % CI		(−1.2)–1.2	(−1.1)–1.1
Adjusted Coeff.	ref	−0.3	0.5
95 % CI		(−1.5)–0.8	(−0.4)–1.4
Conduct problems			
Unadjusted Coeff.	ref	−1	2
95 % CI		(−4.2)–2.2	(−0.8)–4.8
Adjusted Coeff.	ref	−0.5	1.5
95 % CI		(−3.7)–2.7	(−0.6)–3.6

^a^ = significant difference between TMD-pain group and TMD-painfree (*p* < 0.05)

Both unadjusted analysis and adjusted for age, sex, ethnic origin and parental income are presented

### Physical activities and social competence

The mean range for children’s physical activities were within border line clinical range for all three groups, whereas the social competence was within the normal range (Table 4). There were no significant associations between the TMD-pain, TMD-painfree, and non-TMD groups in this respect.

**Table 4:** The range for children’s physical activities and their social competence in 456 randomly selected children and adolescents in the general population of the city of Jeddah, Saudi Arabia

	Non-TMD	TMD-painfree	TMD-pain
Activites			
Mean (SD)	32.6 (7.7)	35.8 (8)	33.4 (7.7)
Median (IQR)	32 (11)	35 (6)	35 (12)
Min-max	20–65	22–61	20–52
Unadjusted	ref	3	1
95 % CI		(−0.4)–6.4	(−2.1)–4.1
Adjusted	ref	2	0.5
95 % CI		(−1.1)–5.1	(−2.5)–3.5
Social			
Mean (SD)	40.5 (7.3)	41.3 (6.6)	41.3 (6.7)
Median (IQR)	41 (11)	43 (10)	41 (10)
Min-max	25–62	28–50	29–59
Unadjusted	ref	2	0
95 % CI		(−2.7)–6.7	(−2.2)–2.2
Adjusted	ref	1	0
95 % CI		(−3.4)–5.4	(−2.5)–2.5

**Fig. 2:**
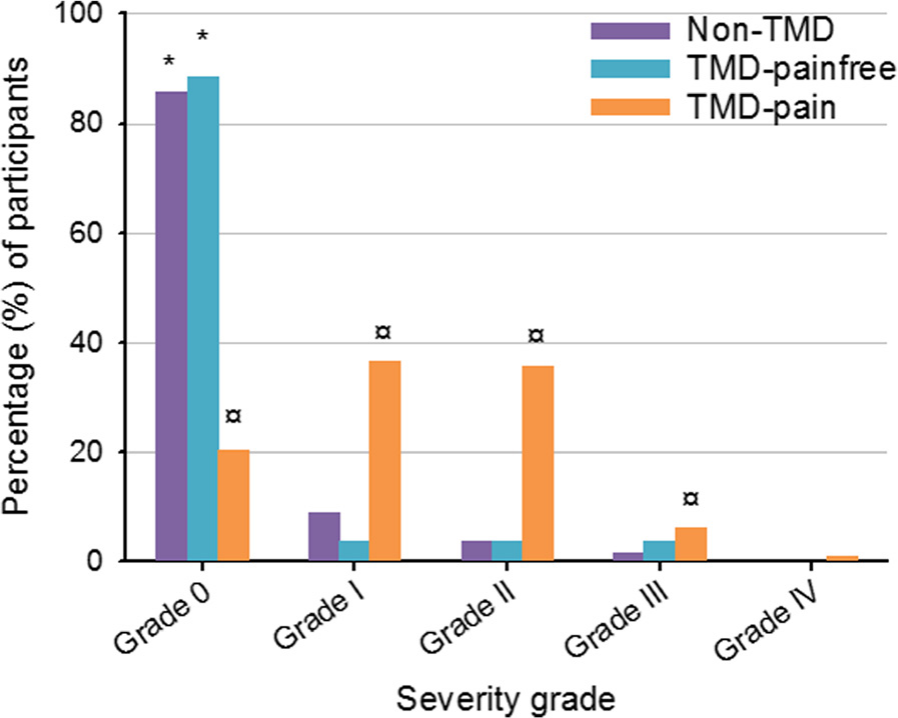
Differences in the severity grades of the Graded Chronic Pain Scale for the three different groups (non-TMD; TMD-pain; TMD-painfree). Children and adolescents in the TMD-pain group scored significantly higher frequencies in the severity grades (i.e., higher than Grade 0) of the Graded Chronic Pain Scale (GCPS) than children and adolescents in the non-TMD and TMD-painfree groups (*p* < 0.05)

According to the GCPS, the children and adolescents in the TMD-pain group scored significantly higher frequencies in the higher severity grades (Grade I, II and III) than the children and adolescents in the non-TMD and TMD-painfree groups (*p* < 0.05), as shown in Fig. 2.

## Discussion

Emotions and/or emotional influences, either pleasant or un-pleasant, play a great role in the pain experience in children and adolescents with a TMD pain condition [27]. Likewise the current study indicates that emotions have a significant association to TMD-pain in children. This since the present study showed significant associations in children and adolescents with a TMD-pain diagnosis and all internalizing problems investigated, i.e., Anxious/Depressed, Withdrawn/Depressed, Somatic Complaints. These findings are coinciding with the findings of previous studies showing that anxiety and depression frequently occur in children and adolescents having TMD signs and/or symptoms [28, 29]. The findings of the present study are also in accordance with other studies, who stated that adolescents with increased TMD-pain had significantly higher frequencies of internalizing problems (i.e., depression, anxiety and somatic complaint) and aggressive behavior compared to healthy controls [7, 18]. This was also reported in a study that showed that children and adolescents who repeatedly visited physicians for their orofacial pain also were complaining of depression in the form of sadness, anger, sleep disturbances as well as problems in school attendance. As a remark that study also reported that myofascial pain in the orofacial region in children and adolescents often is misdiagnosed as recurrent ear infections [30]. An explanation for the increased pain sensation might be due to the fact that anxiety exacerbates the masticatory muscle tension by clenching and grinding, which in turn leads to an increased release of pro-inflammatory cytokines followed by a sensitization of the whole pain pathway [31]. Furthermore, another study reported a significant association between TMD and anxiety [32]. However, in contrast to the present study, that study did not find any association between TMD and depression [32]. One explanation to this difference could be the fact that they included a younger age group (8–12 years) than the present study (10–18 years). Another could be that they have used a different scale that was not validated for children, the Hospital Anxiety and Depression Scale (HADS), to assess depression and anxiety. Concurrently, it has been shown that acute short-term TMD conditions are more frequently associated with anxiety, whereas long-term TMD conditions are more correlated to depressive disorders [33]. Finally, Hofstra and co-workers (2001) followed the same children and adolescents for 10 years and they found that all internalizing problems that children and adolescents reported remained to the adulthood [7]. This finding indicate that some problems remain during the transition stage from childhood to the adulthood and this warrants not only early diagnosis of TMD-pain in children and adolescents but also a decent management of TMD-pain in children.

As in this study, also other studies showed that children and adolescents with TMD-pain report a significantly higher degree of somatic complaints than children and adolescents without TMD pain. However, in contrast to this study they reported that the somatic complaints were more intense than emotional complaints [5, 27]. Moreover, this study also indicates that aggressive behavior and thought problems associated with TMD-pain, which is in similarity with a longitudinal study showing that aggressive behavior among these children and adolescents is passed on to adulthood [34].

The TMD-pain group showed significant associations to affective, somatic and anxiety problems according to the DSM-oriented scale in the present study. In view of this, a previous longitudinal study showed that emotional and behavioral problems in childhood continued to adulthood and met the criteria of DSM-VI [7]. Hence, these affective, somatic and anxiety problems in the TMD-pain group might go on into the adulthood and consequently have a negative impact on the individual’s quality life. One explanation to why emotional and behavioral problems continue to adulthood, when they are associated with TMD-pain, could be that cognitive and nociceptive systems already affected by pain once, easily can recall these associations [27]. This, since the affective problems and their associations with TMD-pain share the pathways of the nervous system that mediate pleasant and unpleasant emotions [35]. Another explanation could be that both sensory and affective abilities share memories from early pain experiences [36]. Further, other studies found that abdominal as well as pain in the temporomandibular region contribute not only to depression and anxiety but also to general body malfunctions and/or somatization [18, 37].

The present study reported limitations in physical activities in both the TMD- and non-TMD groups. However, no significant associations were observed between the groups. However, previous studies have shown a weak relationship between TMD, headache and low incidence of sport activity [18, 38]. Furthermore, the current study shows that social activities (relations) were within the normal range in all groups. Hence, it seems that TMD-pain did not limit their social relations which is comparable to the results of List and co-workers (2001) who found that TMD-pain did not have any effect on daily activities [18].

This study indicates that the risk of developing internalizing problems, such as Anxious/Depressed, Somatic Complaints and Attention Problem, is increased in boys with TMD-pain when compared to girls. This finding is in contrast to a previous study indicating that depressive symptoms and somatic complaints co-occurred in girls more than boys with TMD pain [17]. One explanation to the finding that boys with TMD-pain have an increased risk of developing internalizing problems could be that boys in this study reported the same level of physical activity as girls, which is in contrast to previous studies in Saudi Arabia indicating that boys report a higher degree of physical activitiy than girls [39]. With this in mind, one could speculate if boys suffering from TMD-pain reported lower physical activities than normal due to the TMD-pain or if TMD-pain might lead to a decreased level of physical activity [40]. Another possible reason for this finding could be related to the drop-outs among the boys. Perhaps those boys were more physically active than the participating boys and therefore did not have the time to participate.

The same study also showed that somatic complaints increased with age. However, that finding is in contrast to the present study where no such increase was found. One explanation could be that since emotions play a great role in the pain experience in children and adolescents with a TMD-pain condition [27]. Then, repeated pain or continuous pain or longer duration of a pain condition will most likely induce un-pleasant emotions such as anxiety, depression, somatic, hyperactivity problems and also oppositional defiant problems, which this study showed to be more present in boys with TMD-pain.

One unexpected finding was the fact that the younger age group (10–13) reported a higher degree of affection problems, and a higher level of anxiety than the older age group (14–18). These significant differences between the age groups might be due to the notion that the younger children have a greater tendency to self-evaluate their pain at the extreme level on the pain scale than the older children [41]. Although the settings for boys and girls were equal, despite the fact that all girls were examined in the school nurse’s room using a mobile dental chair and the boys at a dental clinic of the primary health care center, one can consider this issue as a limitation for the study, which might have led to a higher amount of dropouts among the boys. However, a strength of the current study is the randomization of the sample that allow the authors to generalize the results, since it was a representative sample for the city. Another strength is that only one examiner, trained in RDC/TMD, performed the clinical examination of all children. A final strength is that the questionnaires were filled in by the children and/or the adolescents without the influence of their parents/guardians.

## Conclusion

In conclusion, TMD-pain in children and adolescents does not seem to affect the social activities but it seems to have a strong association to emotional, behavior and somatic functioning, with higher frequencies of anxiety, depression, somatic problems, aggressive behavior and thought problems than children and adolescents without TMD-pain. With respect to the biopsychosocial model the present study indicates that there are significant associations to psychosocial, somatic and behavioral comorbidities and TMD-pain in children and adolescents in the Middle East region.

## Abbreviations

DP: Disability Points
GCPS: Graded Chronic Pain Scale
RDC/TMD: Research Diagnostic Criteria for Temporomandibular Disorders
SCL-90-R: Symptom Checklist-90-Revised
TMD: Temporomandibular Disorders
TMJ: Temporomandibular Joint
YSR: Youth Self Report scale
